# Galectin Hco-gal-m from *Haemonchus contortus* modulates goat monocytes and T cell function in different patterns

**DOI:** 10.1186/1756-3305-7-342

**Published:** 2014-07-23

**Authors:** Wang Wang, Shuai Wang, Hui Zhang, Cheng Yuan, RuoFeng Yan, XiaoKai Song, LiXin Xu, XiangRui Li

**Affiliations:** College of Veterinary Medicine, Nanjing Agricultural University, Nanjing, 210095 PR China

**Keywords:** *Haemonchus contortus*, Galectin, PBMC, Monocytes, T cells

## Abstract

**Background:**

Monocytes and T cells are two major subpopulations of peripheral blood mononuclear cells (PBMC) and play an essential role in the innate and adaptive immune systems. Different members of the galectin family show multiple and distinct regulatory effects on different cell types. Previous studies have demonstrated that the galectin from *Haemonchus contortus* (Hco-gal-m) performed immunomodulatory effects on goat PBMC, however, which subpopulation of PBMC is the primary target of Hco-gal-m and whether the immune modulations share the same mechanism remain unclear.

**Methods:**

In this study, the developmental expression of Hco-gal-m was analyzed by RT-PCR and Western blot analysis. The distribution of Hco-gal-m in adult worm was detected by an immunohistochemical test. The binding activity of the recombinant Hco-gal-m (rHco-gal-m) on goat monocytes and T cells were assessed by flow cytometry. The immunomodulatory effects of Hco-gal-m on cytokine secretion, cell activation and apoptosis were observed by co-incubation of rHco-gal-m with goat monocytes and T cells.

**Results:**

Hco-gal-m was expressed in L4 as well as adult worms and predominantly localized at the internal surface of the worm guts. rHco-gal-m could bind to both monocytes and T cells. The engagement of rHco-gal-m decreased the production of IL-6, IL-10 and TNF-α in T cells, however, it significantly increased the secretion of IL-10 in monocytes. After rHco-gal-m exposure, the expression of MHC-II on monocytes and that of CD25 on T cells were restricted. Consequently, T cell proliferations were potently inhibited by rHco-gal-m. In addition, rHco-gal-m induced apoptosis in T cells, but not significantly in monocytes.

**Conclusions:**

Our results indicated that rHco-gal-m modulated goat monocytes and T cell function in different patterns.

**Electronic supplementary material:**

The online version of this article (doi:10.1186/1756-3305-7-342) contains supplementary material, which is available to authorized users.

## Background

Galectins, a family of S-type lectins found in a large scale of species, are characterized by two features: a specific affinity for β-galactoside and a conserved specific sequence motif called carbohydrate recognition domain (CRD) [1]. To date, 15 mammalian galectins (galectin-1 to -15) have been cloned and functionally characterized [2], revealing various roles in apoptosis [3], chemo-attraction [4], cell adhesion [5], cell proliferation [5], cytokine secretion [6] and immune responses [7]. After the discovery of the first nematode tandem repeat type galectin in *Caenorhabditis elegans* (*C. elegans*) [8], similar galectins have also been isolated from a number of helminth parasites including *H. contortus*.

A growing body of experimental evidence indicated that the parasitic galectins played important roles in the parasite-host interaction. A recombinant galectin of *Toxascaris leonina* could enhance the production of TGF-β and IL-10 in mouse spleen lymphocytes and suppress intestinal inflammation [9]. Galectins of *T. colubriformis* were shown be recognized by sera from sheep artificially infected with the nematode [10]. Turner *et al*. [11, 12] reported that galectins extracted from infective larvae (L3 stage) of *H. contortus* exhibited a specific chemokinetic activity to attract eosinophils.

In our previous research, we reported that two isoforms of galectins, Hco–gal-m (Acc. No. AY253330) and Hco–gal-f (Acc. No. AY253331), derived, from male (m) and female (f) *H. contortus*, respectively [13]. Although, two galectins are varied by one amino acid in the C-terminal CRD [14], both rHco–gal-m and rHco–gal-f (rHco-gal-m/f) can induce the same biological effects and work in the same way. They inhibited the hemagglutination of goat erythrocytes [14], suppressed cytokine mRNA transcription [15], induced apoptosis of the goat PBMC [16], and showed partial immunoprotective effects against *H. contortus* infection [17]. Recently, a combined proteomic and transcriptomic analysis revealed that the activations of VEGF pathway, free radical producing pathway, NFκB pathway and ubiquitin–proteasome pathway in goat PBMC were down-regulated by rHco-gal-m/f [18].

PBMC is a mixture of subpopulations of function cells, which includes lymphocytes (T cells, B cells, and NK cells), monocytes, and dendritic cells [19]. All of the subpopulations are critical to the host responses to pathogen infections. Monocytes and T cells are two major subpopulations of PBMC. The best known function of monocytes is as a considerable systemic reservoir of myeloid precursors for the renewal of tissue macrophages and other antigen-presenting cells (APC) [20]. Monocytes are also key effectors of the innate immune response to pathogens and contribute to recruitment of T-cells at sites of infection [21]. In turn, the activated T cells assist other white blood cells in immunologic processes, including maturation of B cells into plasma cells and memory B cells, and activation of macrophages. Once activated, T cells divide rapidly and secrete cytokines that regulate or assist in the active immune response [22].

It was reported that individual galectins could act on multiple cell types and induce various biological effects on different cells [23]. Although we have demonstrated that Hco-gal-m/f has an important immunomodulatory effect on goat PBMC, it is still unclear which subpopulation is the primary target of this immune modulation effect and whether the immune modulations share the same mechanism.

In the current study, by utilizing rHco-gal-m, we further explored the unknown biological characteristics of Hco-gal-m/f and their immunomodulatory effects on goat monocytes and T cells.

## Methods

### Ethics statement

The experiment was conducted following the guidelines of the Animal Ethics Committee, Nanjing Agricultural University, China. All experimental protocols were approved by the Science and Technology Agency of Jiangsu Province. The approval ID is SYXK (SU) 2010-0005.

### Reagents and antibodies

Ficoll-hypaque was obtained from GE Healthcare (Little Chalfont, UK). Electrophoresis reagents were from Bio-Rad (California, USA). RIPA buffer, DAPI and Anti-Fade Fluoromount solution were purchased from Beyotime Institute of Biotechnology (Jiangsu, CHN). Cell culture medium (RPMI 1640), fetal calf serum (FCS), penicillin and streptomycin were from Invitrogen. DTT, Tween-20, 2-mercaptoethanol, phenylmethylsulfonyl fluoride (PSMF), Triton X-100, lipopolysaccharide (LPS) and Concanavalin A (ConA) were purchased from Sigma-Aldrich (Missouri, USA). Goat Anti-Rat IgG H&L (Alexa Fluor® 647, ab150159) was purchased from Abcam (Massachusetts, USA). Goat anti-rat IgG-PE (sc-3740) was purchased from Santa Cruz Biotechnology (California, USA). Monoclonal antibodies MHC-I (MCA2189A647), MHC-II (MCA2226F) and isotype controls were purchased from AbD Serotec (Oxford, UK). CCK-8 was from Dojindo Laboratories (Kumamoto, JPN).

### Parasites

*H.contortus* strain (designated Nanjing 2005) was maintained by serial passage in 3-6-month-old, helminth-free goats [24]. *H. contortus* eggs were recovered according to Hubert and Kerboeuf [25]. The faecal sample was suspended in water and cleared of organic debris by filtration through 1 mm and 150 μm sieves. Eggs were collected on a 25 μm sieve and further cleared of organic debris by centrifugation in magnesium sulphate (density 1.10 g/cm^3^) for five minutes at 1000 × g. The supernatant was filtered through 100 μm sieves and the eggs were washed in water and collected on a 25 μm sieve. Faecal cultures from goats were incubated at 27°C to recover infective third-stage larvae (L3) after 6-7 days [26]. L3 were exsheathed by exposure to 0.2% sodium hypochlorite (NaOCl) bubbled with air for 30 min at room temperature [27]. Exsheathed L3 (xL3) were separated from cuticular casts by migration through two 20 μm nylon meshes. xL3 were axenised in antibiotic solution (0.6 mg/ml penicillin, 1 mg/ml streptomycin, 40 μg/ml gentamycin and 10 μg/ml amphothericin B), then suspended in RPMI 1640 medium containing 20% (v/v) fetal bovine serum placed in culture flasks (175 cm^2^, vented cap, Corning) at a concentration of 1,000-2,000 larvae/ml and incubated at 40°C in 20% CO_2_ for 7 days to produce early fourth-stage larvae (L4) [27]. The presence of a majority of individuals with L4 stage mouthparts, as described by Sommerville [28] and Mapes [29], was confirmed by microscopy. Adult worms were collected at necropsy from the abomasa of infected donor goats 28 days after inoculation with 8,000 L3. *H.contortus* of each stage were washed extensively in phosphate buffered saline (PBS) several times in order to remove any residual and subsequently frozen at -70°C for later use.

Local crossbred male goats (3–6-month-old) from the teaching and research flock at Nanjing Agricultural University were housed indoors in pens containing six goats per pen. The male goats were fed hay and whole shelled corn and provided with water ad libitum. All goats were dewormed twice at 2 week intervals with levamisole (8 mg/kg bodyweight) orally at the time of housing to remove naturally acquired strongylid infection. After 2 weeks, a fecal sample from each goat was examined by microscopy for helminth eggs, according to standard parasitological techniques. Goats exhibiting no eggs were used in the subsequent study and daily health observations were performed throughout the experiment.

SD rats (body weight ~150 g) were purchased from the Experimental Animal Center of Jiangsu, PR China (Qualified Certificate: SCXK 2008-0004) and were raised in a sterilized room and fed sterilized food and water.

### Preparation of rHco-gal-m

The rHco-gal-m was expressed as previously described [14]. In brief, Total RNA was isolated from *H. contortus* adult worms followed by cDNA synthesis. The DNA fragment encoding for Hco-gal-m was PCR amplified and cloned into the pBV220 prokaryotic expression vector. The constructed plasmids were transformed into *Escherichia coli* strain DH5α. The cells containing Hco-gal-m expression plasmid were cultured in Luria-Bertini medium with ampicillin (100 μg/ml) and induced at 42°C to express the recombinant proteins.

The purification of rHco-gal-m was conducted by affinity chromatography. The cell pellet obtained from one liter culture was suspended in 100 ml of wash buffer (10 mM Tris-HCl (pH 7.4) containing 0.5 M NaCl, 5 mM 2-mercaptoethanol, 1 mM EDTA and 1 mM PSMF) and then sonicated for 15 min on ice. The sonicate was supplemented with 1% (w/v) Triton X-100 and then stirred for 30 min at 4°C, followed by centrifugation. The resulting supernatant was added to a lactose-agarose column (Sigma-Aldrich) and allowed to react at room temperature for 2 h. The bound fraction was eluted with 20 mM lactose dissolved in the wash buffer and then dialyzed against PBS (pH 7.4) containing 0.1 mM DTT (PBS/DTT) (Additional file 1: Figure S1).

LPS was depleted from the rHco-gal-m using Detoxi-Gel Affinity Pak prepacked columns (Pierce, USA). The concentrations of the recombinant proteins were equalized to 1 mg/ml prior to LAL assay. Endotoxin levels of the protein samples were measured by LAL gel clot assay using a Pyrosate® Kit (Cape Cod Inc., USA). The samples whose endotoxin content was less than the sensitivity of the Pyrosate kit (<1EU per 1 mg of the recombinant proteins) were collected for the subsequent experiments. The *E. coli* containing empty plasmid were cultured and the cell lysates were purified under the same conditions. The same volume of purified bacterial lysate (PBL) was used as the mock control in some experiments.

### Generation of polyclonal antibody

Rat polyclonal antibodies against rHco-gal-m were prepared as described previously [18]. Briefly, about 0.3 mg of the purified rHco-gal-m was mixed with Freund’s complete adjuvant of a 1:1 mixture and injected into SD rats subcutaneously in multiple places followed the method described by Han *et al*. [30]. After the first injection, rats were then boosted four times at 2-week intervals with the same dose. The serum containing specific anti-rHco-gal-m antibodies was harvested 10 days following the last injection and then stored at -70°C for later use.

### RT-PCR analysis of Hco-gal-m transcription

To determine whether expression of Hco-gal-m is restricted to specific stages of the parasite’s life-cycle a reverse transcriptase PCR (RT-PCR) experiment was carried out. RNA extraction and preparation of cDNA were performed as previously reported [18]. In brief, total RNA was isolated from eggs, L3, xL3, L4 and adult worms using RNeasy Mini Kit (Qiagen, Germany). Then, the RNA was reverse-transcribed at 52°C for 1 h by ThermoScript RT-PCR System (Invitrogen, USA) according to the standard protocol. The RT-PCR utilized cDNA prepared from different life-cycle stages of *H. contortus* as template and specific primers for the complete ORF of Hco-gal-m (852 bp). The primers were designed by primer premier software (version 5.0) and were listed as follows: Hco-gal-Forward (5′-ATGGTGTCACAGTTCCTACACTGGT-3′) and Hco-gal-Backward (5′-CTACTGGATCTGGATGCCAGTCA-3′). The thermal cycle commenced with a hot start at 94°C for 5 min, followed by 30 cycles each consisting of 94°C for 60 s, annealing at 55°C for 60 s, and extension at 72°C for 90 s, and terminated after a final 10 min period at 72°C. The products were separated on a 1.5% agarose gel and visualized by ethidium bromide staining under UV light.

### Western blot analysis of Hco-gal-m expression

Western blot was performed according to the method reported by Arata *et al*. [31]. Briefly, samples including crude somatic extracts of eggs, L3, xL3, L4 and adult worms were electrophoresed on a 12% SDS-PAGE gel. Then, the proteins were electro-transferred onto a nitrocellulose membrane. After being blocked with 5% (w/v) skimmed milk powder in PBST (PBS with 0.5% Tween-20) overnight at 4°C, the membranes were incubated with the primary antibody against rHco-gal-m (dilutions 1:50) for 1 h at 37°C. The membranes were washed 15 min × 3 with PBST and then incubated with the secondary antibody goat anti-rat IgG-HRP (Santa Cruz, USA) in PBST for another 1 h at 37°C. After three washes, the immunoreaction was visualized using ECLsystem (Amersham Biosciences, UK).

### Localization of Hco-gal-m by immunohistochemical study

Washed adult worms suspended in PBS were fixed in 4% formaldehyde-0.2% glutaraldehyde in PBS for 90 min and then immersed in TISSUE-TeK® O.C.T. compound (SAKURA, USA). They were snap frozen in liquid nitrogen and stored at -20°C until required for further processing. Cryostat sections of 10 μm thickness were cut, washed with PBS, and treated for 60 min with 10% normal goat serum in PBS to prevent non-specific binding of antibodies. The sections were then incubated with specific rat anti-rHco-gal-m antiserum (1:100 dilution) or normal rat serum (control) for 60 min at 37°C, washed 15 min × 3 with PBS, and subsequently incubated for 60 min with Goat Anti-Rat IgG H&L Alexa Fluor® 647 (Abcam, UK). Finally, the sections were stained with DAPI (Beyotime Institute of Biotechnology, CHN) to show DNA. After washing with PBS, the specimens were immersed in Anti-Fade Fluoromount solution (Beyotime Institute of Biotechnology, CHN), which prevents fading of fluorescence during microscopic examination.

### Isolation of monocytes and T cells

PBMCs were separated from heparinised blood with the standard Ficoll-hypaque (GE Healthcare, USA) gradient centrifugation method and washed twice in Ca^2+^/Mg^2+^-free PBS pH 7.4. The PBMCs were resuspended to a final density of 1 × 10^6^ cells/ml in RPMI 1640 (Invitrogen, USA) containing 10% heat inactivated fetal calf serum (FCS), 100 U/ml penicillin and 100 mg/ml streptomycin (Invitrogen, USA). To obtain monocytes, the PBMCs were plated in 24-well culture plates and incubated for 2 h at 37°C in 5% CO_2_. The non-adherent cells were aspirated and incubated separately under the same conditions for later use.

T cells were isolated from the non-adherent population. The non-adherent cells were subject to nylon wool columns (Wako, Japan) as previously described [32, 33]. Briefly, the columns were washed and equilibrated with warm (37°C) complete RPMI. 3 × 10^8^ cells were suspended in 2 ml of warm complete RPMI, added into the column and incubated at 37°C for 60 min. The column was eluted with 20 ml of warm complete RPMI and the T cells were recollected in a tube. Viability of monocytes and T cells was > 95% in all the experiments as measured by trypan blue exclusion test.

### Binding of rHco-gal-m to monocytes and T cells

Binding of rHco-gal-m to monocytes and T cells was determined as previously described [34]. Monocytes and T cells were incubated with 5 μg/ml rHco-gal-m or equal volumes of control buffer (PBS/DTT) for 1 h at 37°C, respectively. Cells were then incubated with rat anti-rHco-gal-m polyclonal antibody (1:100 dilution) followed by staining with the secondary antibody goat anti-rat IgG-PE (Santa Cruz, USA). The binding was quantified using a flow cytometer (FACSCalibur; BD Biosciences).

### Detection of cytokine secretion

To determine cytokine secretion, monocytes and T cells were stimulated with LPS (100 ng/ml) or ConA (10 μg/ml) respectively for 72 h in the presence or absence of rHco-gal-m. The supernatants were collected and cytokine testing was performed by ELISA. Based on the known cross-reactivity with goat cytokines [35, 36], the concentrations of TNF-α, IL-6 and IL-10 were assessed with the following commercially available ELISA kits: Bovine TNF-α duo set (R&D System, UK); Bovine IL-6 ELISA kits (Thermo Scientific, USA) and Bovine Interleukin 10 (Cusabio Biotech Co., LTD.).

### Analysis of MHC molecule expression

The purified monocytes (0.5 × 10^6^ cells/ml) were incubated with different concentrations of rHco-gal-m or equal volumes of control buffer for 24 h in complete RPMI 1640 at 37°C. Cells were then stained with the monoclonal antibodies to MHC-I (MCA2189A647) and MHC-II (MCA2226F), and analyzed on a FACS Calibur cytometer (BD Biosciences). Results were expressed as the percentage of mean fluorescence intensity (MFI) of control.

### Cell proliferation assays

T cells (1 × 10^6^ cells/ml) were activated with ConA (10 μg/ml) and incubated at the same time with a serial concentrations of rHco-gal-m or equal volume of control buffer at 37°C and 5% CO_2_ for 72 h. CCK-8 solutions (Dojindo, Japan) were added to each well of the plates 4 h before harvesting and the absorbance values at 450 nm (OD_450_) were measured using a microplate reader (Thermo Scientific, USA). Cells exposed to ConA with control buffer served as controls and the OD_450_ in controls were set as 100%. Cell proliferation index was calculated by the formula: OD_450_ rHco-gal-m /OD_450_ control.

### Real-time PCR analysis of the marker genes

T cells were activated with ConA (10 μg/ml) and simultaneously treated with rHco-gal-m (20 μg/ml) or equal volumes of control buffer at 37°C and 5% CO_2_ for 24 h. The expression of CD25, CCNA1, CCNB1 and CCND1 was analyzed by real-time PCR. RNA extraction and preparation of cDNA were performed as previously reported [18]. Real-time PCR was conducted on the ABI 7500 Real-Time PCR System (Applied Biosystems, USA), using standard procedure (Stage 1: Initial denaturation, 95°C for 30 s, 1 cycle; Stage 2: Amplification, 95°C for 5 s, 60°C for 60 s, 40 cycles; Melt Curve Stage: 60°C–95°C, ramp rate 1%). Each reaction was performed in a total volume of 20 μl containing 1× SYBR® Premix Ex Taq (TaKaRa, Japan), 500 nM of each primer and a constant amount of cDNA (corresponding to 20 ng of reverse transcribed RNA for each sample). To ensure cDNA samples were not contaminated with genomic DNA, reactions were set up using 20 ng of non-reverse transcribed RNA in place of cDNA. Failure to generate a detectable signal signified the sample as DNA free. Negative (no template) controls were included in each PCR run. Melt curves were generated to ensure a single amplicon had been produced. Primers specific for target goat genes were designed with Beacon Designer 7.0 software (Premier Biosoft International, USA) according to manufacturer’s guidelines and the efficiency of each primer set was verified by running standard curves in triplicates using serial dilutions of the cDNA (Table 1). The amplification efficiency was calculated from the slope of the standard curve by the formula: E = 10^-1/slope^ for each run and ranged between 90% and 110%. Target gene expression was normalized to GAPDH, using the 7500 software version 2.0.6 (Applied Biosystems, USA). T cells exposed to ConA with control buffer were selected as the reference sample and the relative mRNA expression levels of target genes in rHco-gal-m treated T cells were calculated using the 2^-ΔΔCt^ method [37]. Each experiment was tested in triplicate.

**Table 1:** **Primer sequences of target genes in real-time PCR assay**

Gene	GenBank	Forward primer	Reverse primer	Amplicon
Name	Accession	5′ → 3′	5′ → 3′	Size (bp)
CD25	NM_174358	AGATGTCCTGGCTTGAAC	TGGATATAGACCTGCTAATACC	134
CCNA1	XM_005687507	CGCCACATTTACAGGCTATTCATT	GTACTTCTCCCTGATTGCTTGCTG	112
CCNB1	XM_005694606	TTGATGGAACTGACTATGCTGGACT	CAGGTAATGCTGTAGAGTTGGTGTC	132
CCND1	XM_005700049	AGGAGGTGAGGGTGGAAGTGTG	GAAGTCATCGGTAGCAGCGAATAA	100
GAPDH	DQ152956	CCTGGAGAAACCTGCCAAGT	GCCAAATTCATTGTCGTACCA	200

### Apoptosis assay

Cell apoptosis was analyzed as previously described using a flow cytometer [16]. In brief, monocytes and T cells were cultured for 24 h in the presence or absence of rHco-gal-m at different concentrations followed by staining with annexin V and PI (eBioscience, USA) according to the manufacturer’s instructions.

### Statistical analysis

Data are expressed as mean ± the standard deviation of the mean. Statistical analysis for significant differences was performed using an analysis of variance, the Student’s *t* test for parametric samples (GraphPad Prism, USA).

## Results

### Developmental regulation of Hco-gal-m expression

**Figure 1:**
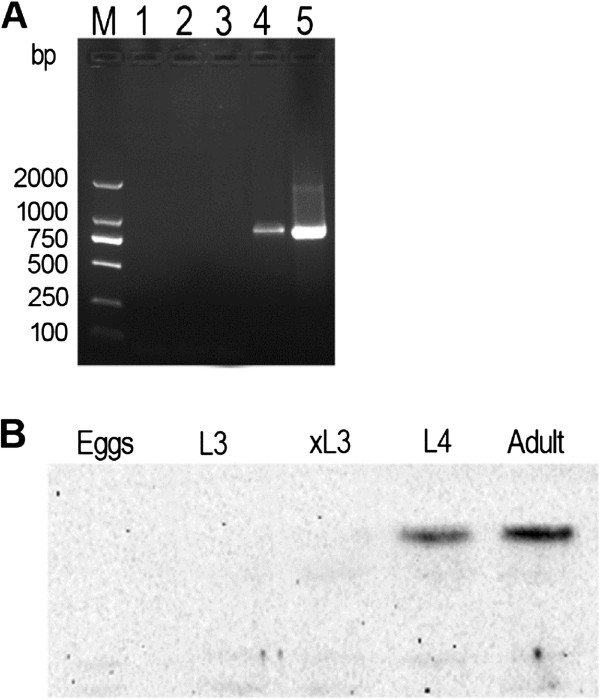
**The timing and site of Hco-gal-m expression.** Typically, the onset of expression of Hco-gal-m is associated with the fourth larval stage and continues in the adults, which were analyzed by RT-PCR **(A)** and western blot **(B)**. M, DL2000 marker; 1, eggs; 2, L3; 3, exsheathed L3; 4, L4; 5, day 28 adults.

RT-PCR analysis showed that there was no significant transcription in eggs and L3, but that a high-steady level of transcription was detected from L4 to adult parasites (Figure 1A). In addition, a similar result was also observed on protein level, which was analyzed by western blot using rat antiserum eluted from rHco-gal-m (Figure 1B).

### Immunolocalization of Hco-gal-m

**Figure 2:**
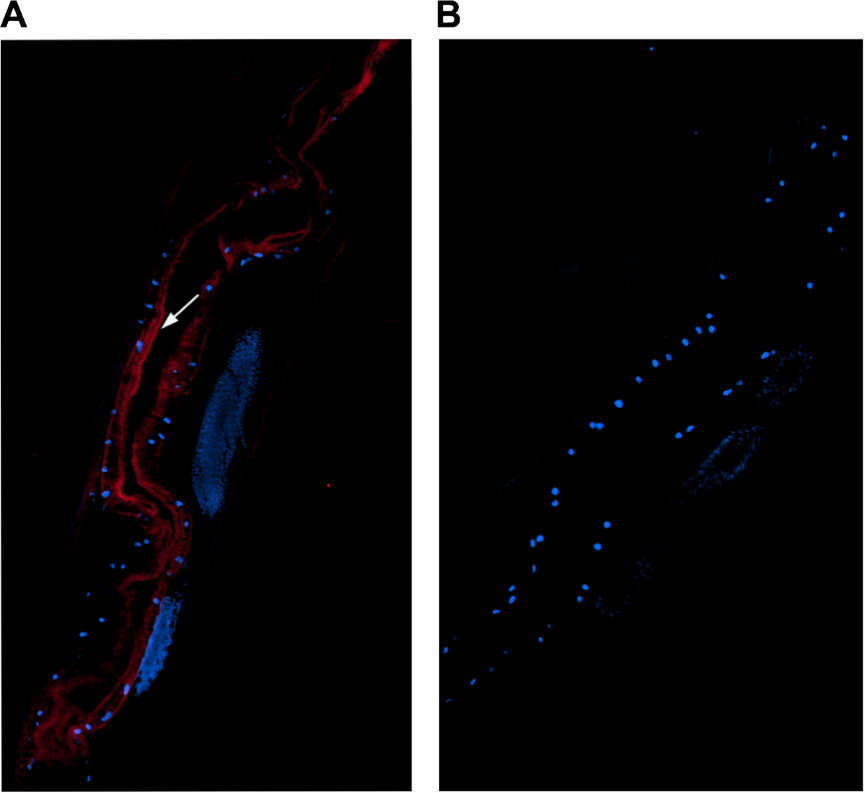
**Immunohistochemical localization of Hco-gal-m protein in cryostal section of** ***H. contortus*** **.** Hco-gal-m protein was detected by the indirect immunofluorescence method using second antibody Goat Anti-Rat IgG H&L (Alexa Fluor® 647). The section was counterstained with DAPI to show DNA. **(A)** Hco-gal-m is localized in the luminal surface of the adult worm’s gut. **(B)** No labeling was observed in negative control. The arrow indicates the gut brush border. Original magnifications: ×400.

A section through a partial body length of an adult female worm was shown in Figure 2. Hco-gal-m and DNA fluoresced red and blue, respectively. Clusters of blue spots observed inside the adult indicating the position of cell nuclei were mainly cross-sections of eggs. The antibody eluted from rHco-gal-m bound predominantly to the internal surface of the parasite’s gut (Figure 2A) and no labeling was observed in control experiments (Figure 2B).

### Binding of rHco-gal-m to monocytes and T cells

**Figure 3:**
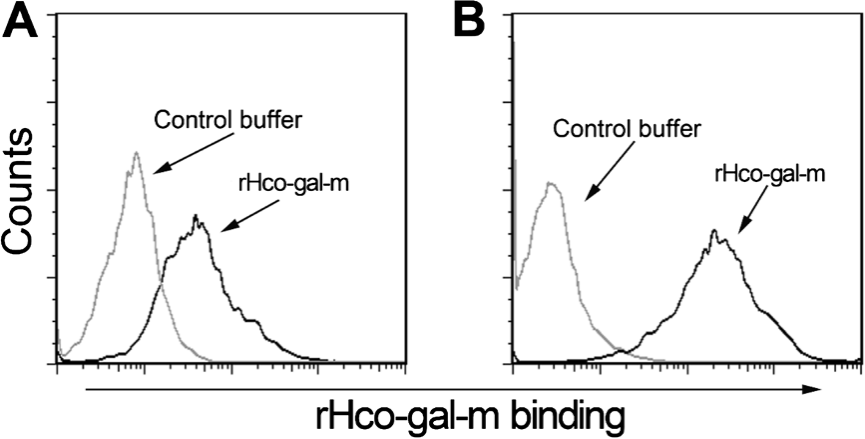
**Binding of rHco-gal-m to monocytes and T cells.** Flow cytometric analysis of rHco-gal-m binding to goat peripheral monocytes **(A)** and T cells **(B)** using rHco-gal-m detected by rat anti-rHco-gal-m antibody and PE-labeled secondary antibody. The data are representative of three individual experiments.

Goat monocytes and T cells were isolated and incubated with rHco-gal-m. The binding of rHco-gal-m to the cells was quantified by flow cytometric analysis. As depicted in Figure 3, rHco-gal-m could bind strongly to both monocytes (Figure 3A) and T cells (Figure 3B).

### Distinct cytokine secretion of monocytes and T cells induced by rHco-gal-m

**Figure 4:**
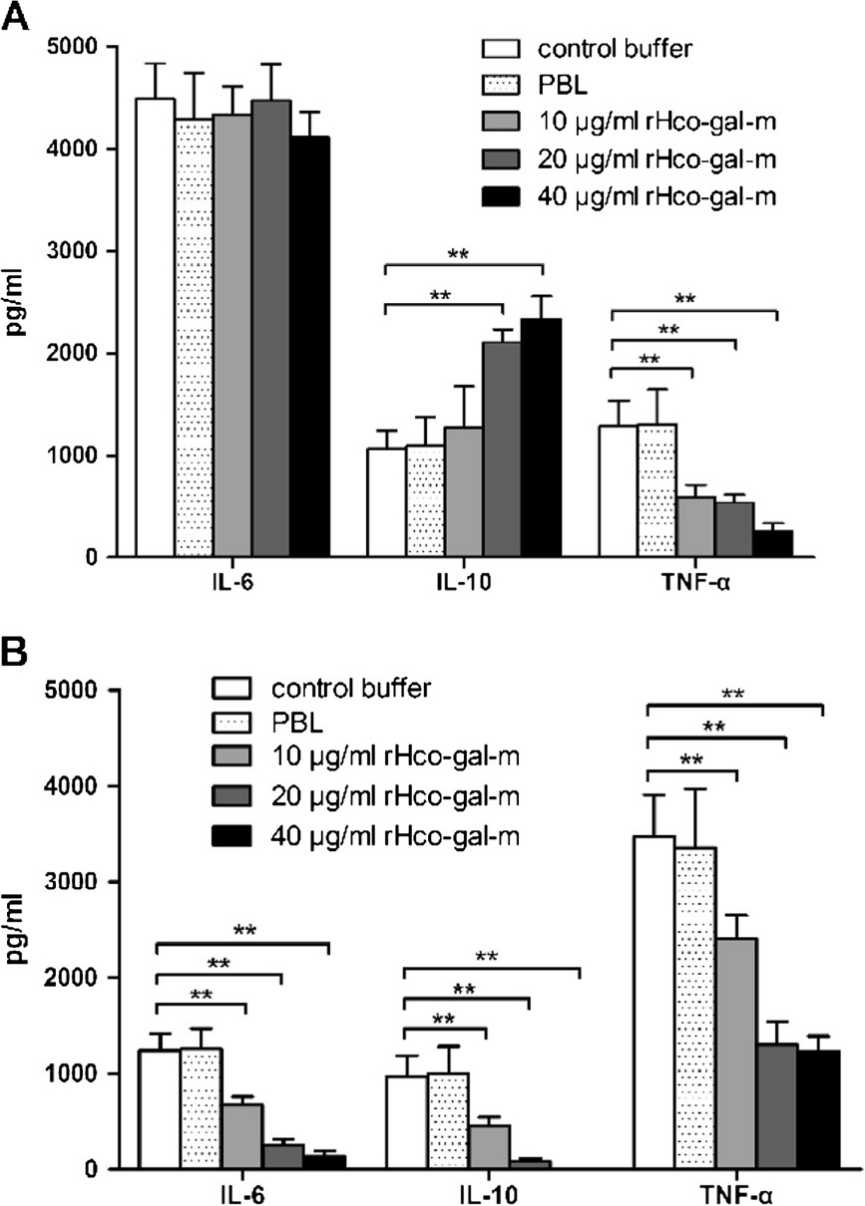
**Distinctive modulation of cytokine secretion by rHco-gal-m.** Monocytes **(A)** and T cells **(B)** were stimulated with LPS (100 ng/ml) or ConA (10 μg/ml) respectively for 72 h in the presence or absence of rHco-gal-m. Cytokine secretion in the supernatant of cell cultures was quantified by ELISA. The data are representative of three independent experiments (**p < 0.01).

By performing ELISA we noted that rHco-gal-m decreased the production of TNF-α in both monocytes and T cells (Figure 4). Interestingly, the secretion of IL-10 was increased in monocytes (Figure 4A) but dramatically inhibited by rHco-gal-m in T cells (Figure 4B). In T cells, rHco-gal-m potently reduced IL-6 (Figure 4B), however, it failed to be significant for IL-6 in monocytes (Figure 4A).

### rHco-gal-m inhibited MHC-II expression on goat monocytes

**Figure 5:**
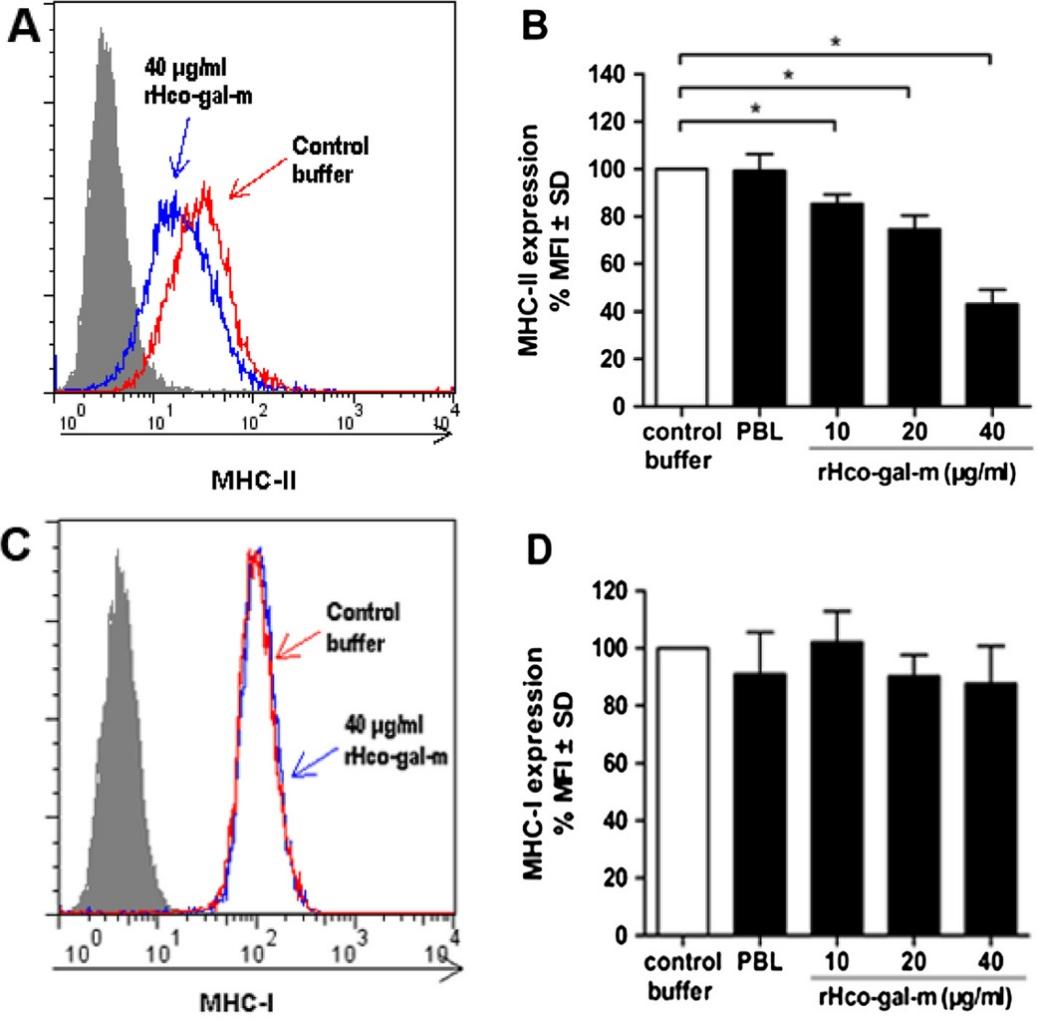
**rHco-gal-m inhibits MHC-II expression on goat monocytes.** Monocytes were cultured in the presence of control buffer (PBS/DTT) or different concentrations of rHco-gal-m for 24 h. MHC-II expression was measured by flow cytometric analysis and calculated as the percentage of mean flourscence intensity (MFI) of controls. Histograms **(A/C)** correspond to 1 representative of three independent experiments, and nonspecific binding was determined using a control isotype antibody (filled histogram). Bars **(B/D)** represent the MFI ± SD of controls. The data are representative of three independent experiments (*p < 0.05).

Compared to the baseline expression of MHC-II in the control buffer, rHco-gal-m significantly decreased MHC-II expression in a dose-dependent manner (Figure 5A,B). However, no changes were detected in MHC-I following exposure of goat monocytes to rHco-gal-m at different concentrations (Figure 5C,D).

### rHco-gal-m inhibited T cells activation and proliferation

**Figure 6:**
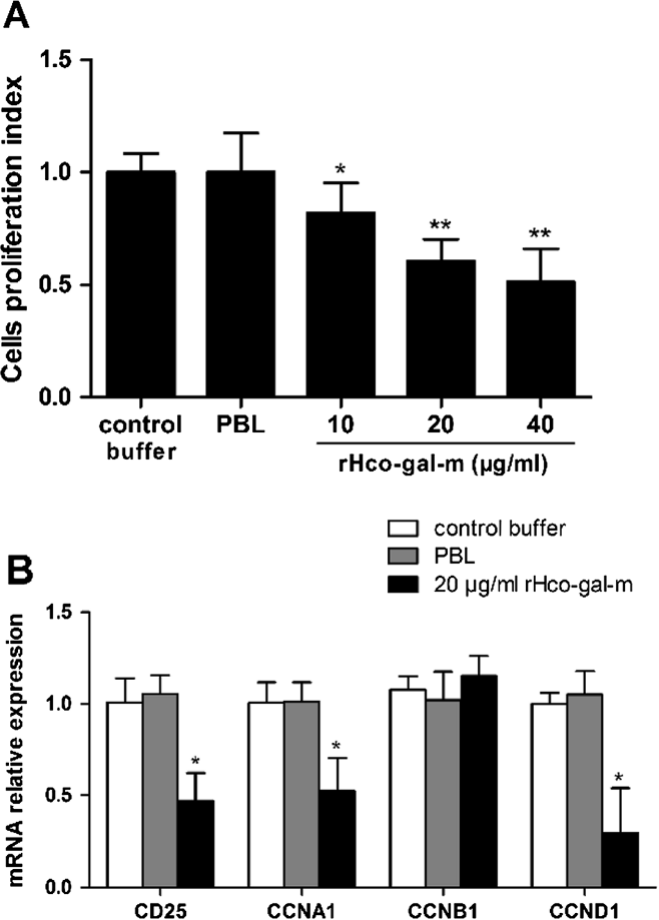
**Inhibitory effect of rHco-gal-m on T cell activation and proliferation. (A)** T cells were activated with ConA and incubated at the same time with serial concentrations of rHco-gal-m at 37°C and 5% CO_2_. The proliferation was measured by CCK-8 incorporation after 72 h. Cell proliferation index was calculated considering the OD_450_ values in controls as 100%. **(B)** T cells were simultaneously treated with ConA and rHco-gal-m at 37°C and 5% CO_2_ for 24 h. The expression of CD25, CCNA1, CCNB1 and CCND1 was analyzed by real-time PCR. All experiments were set up in triplicate. The data are representative of three independent experiments (*p < 0.05 and **p < 0.01).

As demonstrated by incorporation of CCK-8, rHco-gal-m significantly inhibited the proliferation of T cells *in vitro* (Figure 6A). This inhibitory effect was further supported by the expression of some marker genes. Real-time PCR analyses proved that, rHco-gal-m significantly inhibited the transcription of CD25, a sensitive marker for T cell activation, and the cell cycle genes, CCNA1 (cyclin A1) and CCND1 (cyclin D1) (Figure 6B).

### rHco-gal-m induced apoptosis of T cells but not monocytes

**Figure 7:**
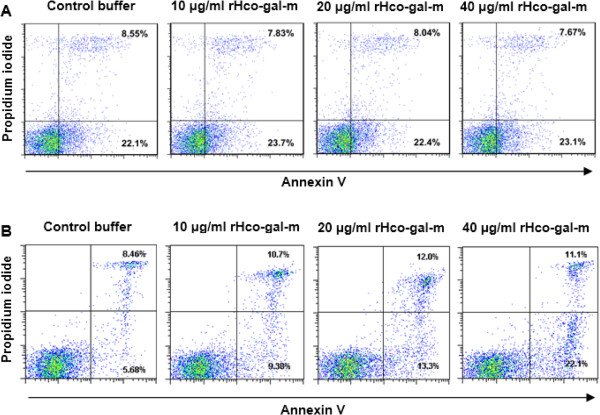
**rHco-gal-m distinctively influences the survival of goat monocytes and T cells.** Goat monocytes and T cells were cultured in the presence of control buffer (PBS/DTT) or different concentrations of rHco-gal-m for 24 h. Apoptosis of monocytes **(A)** and T cells **(B)** was determined by staining with annexin V and PI followed by flow cytometric analysis. Data are representative of three individual experiments.

Using the externalization of membrane phosphaidylserine (PS) as a marker of cell apoptosis and positive DNA staining as an indicator for membrane leakage, it was observed that there was no significant change in annexin V positive monocytes when treated with control buffer or increasing concentrations of rHco-gal-m (Figure 7A). On the contrary, rHco-gal-m induced T cell apoptosis in a dose-dependent manner (Figure 7B).

## Discussion

Hco-gal-m has a tandem repeat structure. Its predicted amino acid sequence shows a high degree of identity with Hco-gal-3b [13] as well as mammalian galectin-4 (Additional file 2: Table S1). In the whole life cycle stages of *H. contortus*, there is no detectable expression of Hco-gal-m in eggs or L3. However, this galectin is expressed in L4 and adults, the more mature stages of the parasite’s life-cycle (Figure 1). The expression coincided with the onset of blood feeding, suggesting that its major role was in the adult parasite, such as development and (or) immune evasion.

Mammalian galectins have been detected in many types of cells and tissue, however, the digestive tract is particularly rich in galectins [38]. Similar to its mammalian homologues, we demonstrated that the native Hco-gal-m protein was predominantly covering the worm’s gut internal surface (Figure 2). In *C. elegans*, a 32 kDa galectin was found to be localized most abundantly in the adult cuticle and the terminal bulb of the pharynx [31]. Hco-GAL-2, a galectin of *H. contortus*, was found to be present on the intestinal brush-border [39]. Proteomic analysis of excretory/secretory (ES) proteins indicated galectins were expressed numerously in *H. contortus*, *Ostertagia ostertagi* and *Teladorsagia circumcincta* [40]. Recently, we found that rHco-gal-m/f could be recognized by the antiserum from goats experimentally infected with *H. contortus* and interacted with goat PBMC *in vitro* [18]. All of these results indicated that galectins of *H. contortus* were excretory/secretory antigens and interacted with the host immune system during infection. Theoretically, the immunomodulatory functions proposed require constant secretion and certain concentrations of this molecule. However, how Hco-gal-m accumulates to the functional concentration *in vivo* and the real mechanism through which it works during natural infection of *H. contortus* should be further studied.

Parasitic galectins have evidenced an increased expression during the parasite infection and have therefore been considered as key players in parasite-host interactions [11, 41]. In this study, rHco-gal-m bound strongly to goat monocytes as well as T cells (Figure 3), and this binding may trigger a cascade of transmembrane signaling events in different biological processes such as activation and homeostasis of host immune cells [2].

Cytokine secretion is a major role of immune cells, enabling communication as well as regulation of the immune system. In a previous study, we found that the activations of VEGF pathway, free radical producing pathway, NFκB pathway and ubiquitin–proteasome pathway in goat PBMC were down-regulated [18] and the cytokine transcription in goat PBMC was inhibited by rHco-gal-m/f [15]. In the present research, rHco-gal-m decreased IL-6, IL-10 and TNF-α secretion in T cells (Figure 4B), but increased the secretion of IL-10 and inhibited the production of TNF-α in monocytes (Figure 4A). These results obviously suggested that rHco-gal-m induced a distinct cytokine secretion pattern in monocytes and T cells and highlighted the multiple and distinct biological effects of galectins on different cell types.

MHC-II molecules are constitutively expressed on the surface of APCs, enabling them to present extracellular antigens and initiate the adaptive immune response [42]. Activation of APCs increases MHC-II expression [43]. Barrionuevo *et al.* [44] reported that galectin-1 inhibited constitutive and inducible MHC-II expression on human monocytes and interfered with MHC-II-dependent antigen presentation. Recently, it was reported that galectins prevented *Salmonella*-induced MHC-II up-regulation and modulated APC activation [45]. In the present study, we noted that rHco-gal-m was able to inhibit MHC-II expression on monocytes in a dose dependent manner (Figure 5). This may due to the "deactivation" of monocytes triggered by high amounts of IL-10 [46]. But, the real mechanisms need further investigations. The main function of MHC-I is to display intracellular proteins to cytotoxic T cells [47]. No significant change of MHC-I expression was observed after exposure of rHco-gal-m in the present study. It might be that *H. Contortus* is a kind of extracellular parasite and Hco-gal-m does not affect the endogenous antigen presentation pathway. However, further studies are required to identify the actual mechanisms responsible.

In this study, incorporation of rHco-gal-m significantly inhibited mitogen-induced activation and proliferation in T cells (Figure 6). T cell activation, cytokine secretion and cell cycling, a complexly regulated movement, was ultimately linked [48]. Cyclin A1 and cyclin D1, encoded by the CCNA1 and CCND1 gene, regulate the S-phase and promote the G1/S transition [49]. Cyclin B1 is encoded by the CCNB1 gene and contributes to the G2/M transition [50]. In this study, the transcription of CCNA1 and CCND1 were decreased (Figure 6B). This suggested that a block in the cell cycle at the G1 phase was induced by rHco-gal-m.

It was reported that rHco-gal-m/f could induce apoptosis of PBMC of goat [16]. In our study, we revealed that goat T cells showed significantly higher susceptibility to rHco-gal-m-induced apoptosis than monocytes (Figure 7), adding more evidence to the fact that different immune cell types can display different phenotypes following exposure to galectins [51–54]. It also indicated that the induction of T cell apoptosis, together with the inhibition of T cell proliferation identified in this study might be one of the mechanisms of *H. contortus* to escape host immune responses.

However, many types of galectins have been reported up to now. But the influence they exert on goat immune cells are seldom studied. This is the first study on the effects of galectin on goat monocytes and T cells. Whether the immunomodulatory properties described here are a specific property of rHco-gal-m or a generic property of parasitic nematode galectins still needs further research.

## Conclusion

In this study, we analyzed the timing and site of Hco-gal-m expression in *H. contortus* and demonstrated that rHco-gal-m could bind to monocytes as well as T cells, and therefore modulated their activation, cytokine production and apoptosis in different patterns. This result provides more evidence in support of the fact that Hco-gal-m is one of the host immunomodulation related molecules and plays an important role in host-parasite interactions. Our study provides a better understanding of the role of this parasitic galectin within the host immune system. However, the specific receptors on the cell surface and the different mechanisms through which rHco-gal-m modulates cytokine secretion, MHC expression and cell apoptosis of T cells and monocytes remain unclear and further studies are required.

## Electronic supplementary material

Additional file 1: Figure S1: The purification of rHco-gal-m. Protein samples were resolved by SDS–PAGE on 12% polyacrylamide gels and stained with Coomassie brilliant blue R250. M: standard protein molecular marker; lane 1: soluble extract of cultured cells; lane 2: the unbound fraction after lactose-agarose affinity chromatography; lane 3: elution of the bound components after lactose-agarose affinity chromatography; lanes 4-7: different concentrations of purified rHco-gal-m after dialysis against PBS/DTT. (DOCX 738 KB)

Additional file 2: Table S1: Similarity of amino acid sequences of Hco-gal-m to various galectins of human, mouse, rat, cattle, sheep and goat. (DOCX 17 KB)
