# Supplementary material for: Galectin Hco-gal-m from Haemonchus contortus modulates goat monocytes and T cell function in different patterns
Source: Parasit Vectors. 2014 Jul 23;7:342. doi: 10.1186/1756-3305-7-342 (PMC4117971; doi:10.1186/1756-3305-7-342)
Supplement: Supplementary file 1 — Additional file 1: Figure S1: The purification of rHco-gal-m. Protein samples were resolved by SDS–PAGE on 12% polyacrylamide gels and stained with Coomassie brilliant blue R250. M: standard protein molecular marker; lane 1: soluble extract of cultured cells; lane 2: the unbound fraction after lactose-agarose affinity chromatography; lane 3: elution of the bound components after lactose-agarose affinity chromatography; lanes 4-7: different concentrations of purified rHco-gal-m after dialysis against PBS/DTT. (DOCX 738 KB) [file 13071_2014_1530_MOESM1_ESM.docx]

**
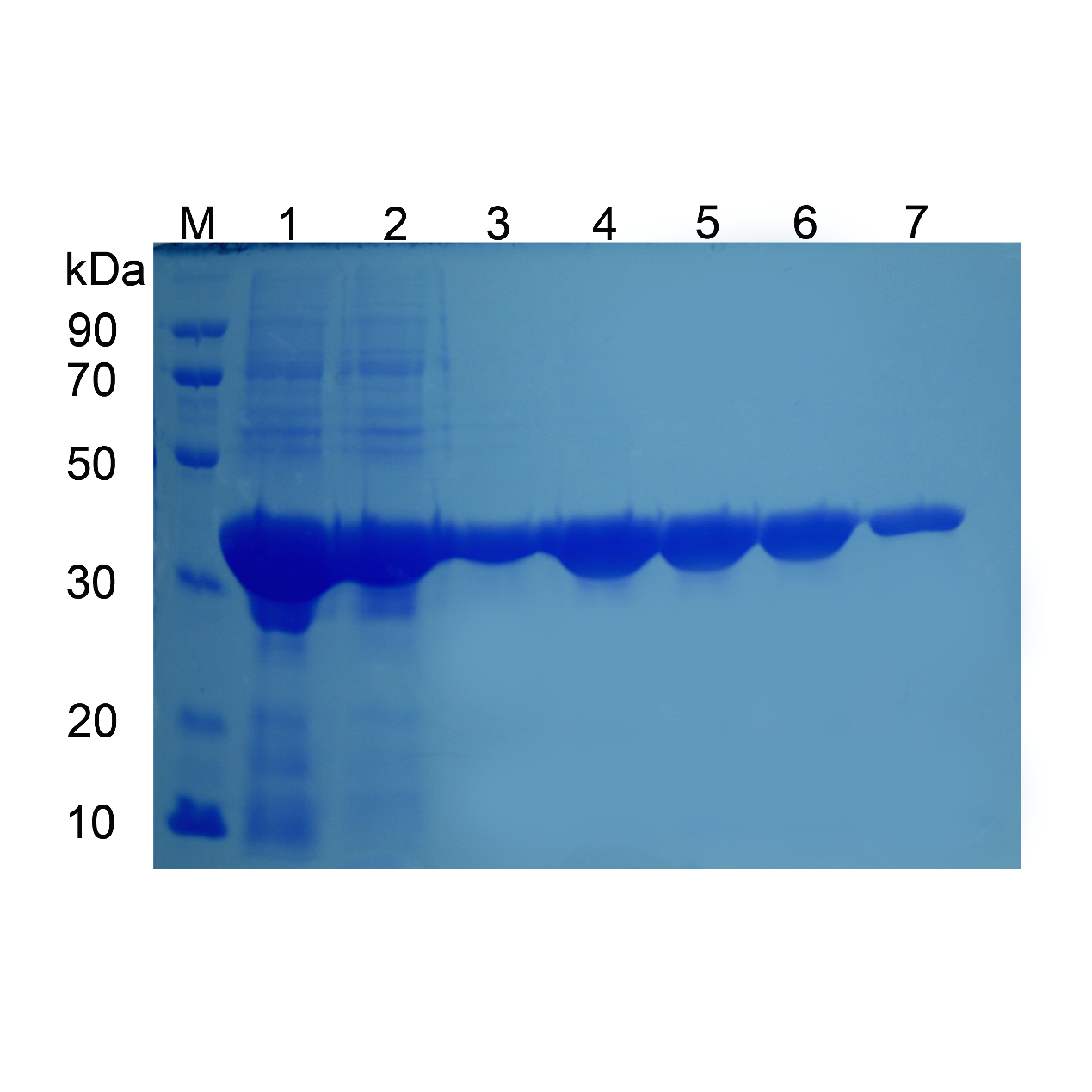
Fig. S1. The purification of rHco-gal-m.**

Protein samples were resolved by SDS–PAGE on 12% of polyacrylamide gel and stained with Coomassie brilliant blue R250. M: standard protein molecular marker; lane 1: soluble extract of cultured cells; lane 2: the unbound fraction after lactose-agarose affinity chromatography; lane 3: elution of the bound components after lactose-agarose affinity chromatography; lanes 4-7: different concentrations of purified rHco-gal-m after dialysis against PBS/DTT.
