# Supplementary material for: Galectin Hco-gal-m from Haemonchus contortus modulates goat monocytes and T cell function in different patterns
Source: Parasit Vectors. 2014 Jul 23;7:342. doi: 10.1186/1756-3305-7-342 (PMC4117971; doi:10.1186/1756-3305-7-342)
Supplement: Supplementary file 2 — Additional file 2: Table S1: Similarity of amino acid sequences of Hco-gal-m to various galectins of human, mouse, rat, cattle, sheep and goat. (DOCX 17 KB) [file 13071_2014_1530_MOESM2_ESM.docx]

**Table S1** Similarity of amino acid sequences of Hco-gal-m to various galectins of human, mouse, rat, cattle, sheep and goat.

| % | Gal-1 | Gal-2 | Gal-3 | Gal-4 | Gal-5 | Gal-6 | Gal-7 | Gal-8 | Gal-9 | Gal-12 | Gal-13 | Gal-14 | Gal-15 |
| --- | --- | --- | --- | --- | --- | --- | --- | --- | --- | --- | --- | --- | --- |
| Human | 15.95 | 16.61 | 26.47 | 34.49 | -* | - | 21.26 | 28.72 | 33.72 | 20.22 | 16.78 | 16.45 |  |
| Mouse | 16.28 | 18.27 | 25.16 | 34.87 | - | 26.26 | 18.27 | 29.85 | 34.59 | 22.92 | - | - |  |
| Rat | 15.95 | 16.94 | 25.49 | 34.78 | 26.40 | - | 18.27 | 31.94 | 33.53 | 23.21 | - | - |  |
| Cattle | 16.28 | 15.28 | 25.16 | 33.24 | - | - | 21.93 | 29.95 | 31.70 | 21.43 | - | - | 17.28 |
| Sheep | 16.61 | 15.28 | 26.47 | 33.82 | - | - | 22.26 | 31.02 | 31.30 | 21.14 | - | 18.73 | 16.94 |
| Goat | - | - | - | - | - | - | - | - | - | - | - |  | 16.61 |

* This subtype of galectin have been not detected.
